# Crystal structures of the gold NHC complex bis­(4-bromo-1,3-di­ethyl­imidazol-2-yl­idene)gold(I) iodide and its 1:1 adduct with *trans*-bis­(4-bromo-1,3-diethyl-imidazol-2-yl­idene)di­iodido­gold(III) iodide

**DOI:** 10.1107/S2056989021011488

**Published:** 2021-11-09

**Authors:** Rolf Büssing, Ingo Ott, Peter G. Jones

**Affiliations:** a Institut für Medizinische und Pharmazeutische Chemie, Technische Universität Braunschweig, Beethovenstr. 55, D-38106 Braunschweig, Germany; b Institut für Anorganische und Analytische Chemie, Technische Universität Braunschweig, Hagenring 30, D-38106 Braunschweig, Germany

**Keywords:** crystal structure, gold, halogen bonds, *N*-heterocyclic carbene

## Abstract

The first title compound, [Au(C_7_H_11_BrN_2_)_2_]I, crystallizes in the space group *P*  without imposed symmetry. The cations and anions are linked to form chains by Br⋯I⋯Br halogen-bond linkages. The second title compound, [Au(C_7_H_11_BrN_2_)_2_][AuI_2_(C_7_H_11_BrN_2_)_2_]I_2_, is an adduct of the first and its formally I_2_-oxidized Au^III^ analogue. It also crystallizes in space group *P*  , whereby both gold atoms occupy inversion centres. The extended structure is a reticular layer involving Br⋯I⋯Br and I⋯I⋯Au linkages.

## Abstract 2

In the first title compound, [Au(C_7_H_11_BrN_2_)_2_]I, the cations and anions form chains *via* halogen bond linkages Br⋯I⋯Br. The second title compound, [Au(C_7_H_11_BrN_2_)_2_][AuI_2_(C_7_H_11_BrN_2_)_2_]I_2_, forms a layer structure involving Br⋯I⋯Br and I⋯I⋯Au linkages.

## Chemical context

**Figure 1:**
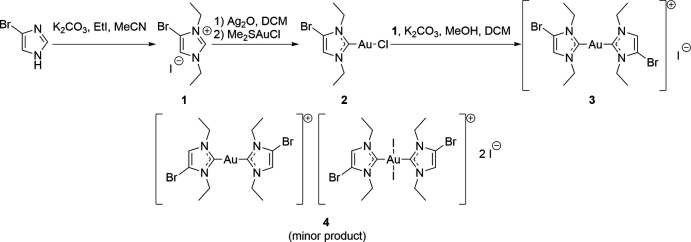
Synthesis of compound **3**, recrystallization of which also afforded a small amount of separable crystals of compound **4**.

Gold complexes have been used in medicine since ancient times and have been applied as drugs for the treatment of rheumatoid arthritis since the 1930s. Currently, gold species are being actively investigated in inorganic medicinal chemistry as possible anti­cancer agents or anti-infectives (Mora *et al.*, 2019 ▸). Some of the existing therapeutics have reached the clinical trial stage as a result of drug repurposing efforts. Metal *N*-heterocyclic carbene (NHC) complexes in general have also proved to be biologically and medicinally active compounds (Ott, 2020 ▸); in particular, gold complexes with NHC ligands are often synthesized and investigated because of the high stability of the gold–carbon bonds and the convenient synthetic access to a broad variety of structurally diverse NHC structures (Nahra *et al.*, 2021 ▸). We have reported on the synthesis, characterization and biological effects of [bis­(4-bromo-1,3-diethyl-imidazol-2-yl­idene)gold(I)] iodide (**3**) (Schmidt *et al.*, 2017*a* ▸) (Fig. 1 ▸). Notably, this complex and related derivatives triggered cytotoxicity against cancer cells, showed a low serum protein binding, and inhibited growth of some pathogenic bacteria. Furthermore, we have recently investigated various gold NHC complexes as anti­bacterial agents and inhibitors of bacterial thio­redoxin reductase (Büssing *et al.*, 2021 ▸). 
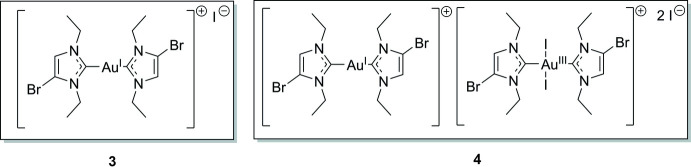


Here we report the structure of **3**, together with that of its 1:1 complex (**4**) with *trans*-[bis­(4-bromo-1,3-diethyl-imidazol-2-yl­idene)di­iodido­gold(III)] iodide, formally its I_2_-oxidized Au^III^ analogue; the latter was formed in small qu­anti­ties when **3** was recrystallized. Further studies on the bioinorganic and medicinal chemistry of **3** and related derivatives are the subject of ongoing projects.

## Structural commentary

**Figure 2:**
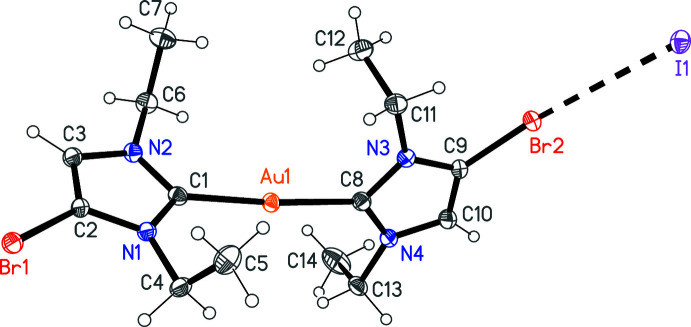
Structure of the asymmetric unit of compound **3**; ellipsoids represent 50% probability levels. The dashed line indicates a halogen bond.

The structure of the asymmetric unit of **3** is shown in Fig. 2 ▸. All atoms lie on general positions in space group *P* 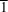 . Selected intra- and inter­molecular dimensions (including contact distances) are presented in Table 1 ▸. The gold atom is, as expected, linearly coordinated. The NHC planes subtend an inter­planar angle of 78.74 (10)°. The short contact Br2⋯I1 seen in Fig. 2 ▸ is one of two such contacts that determine the crystal packing (see next section).

**Figure 3:**
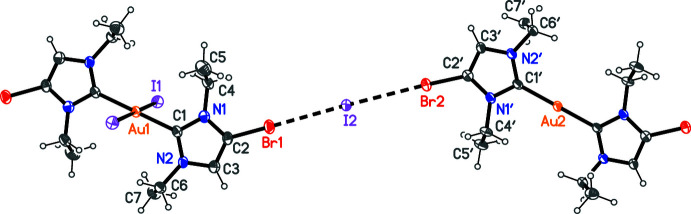
Structure of compound **4**; the asymmetric unit has been extended by symmetry to show complete cations. Ellipsoids represent 50% probability levels. The dashed lines indicate halogen bonds.

The structure of compound **4** is shown in Fig. 3 ▸. Selected metrical parameters for intra- and inter­molecular inter­actions (including contact distances) are presented in Table 2 ▸. Both gold atoms lie on inversion centres; the C—Au—C and I—Au—I angles are thus exactly linear, and the NHC planes of both cations are exactly coplanar. The gold(III) centre displays the expected square planar geometry. The Au—C bond is slightly longer than in **3**. For further discussion, see *Database survey* below.

## Supra­molecular features

**Figure 4:**
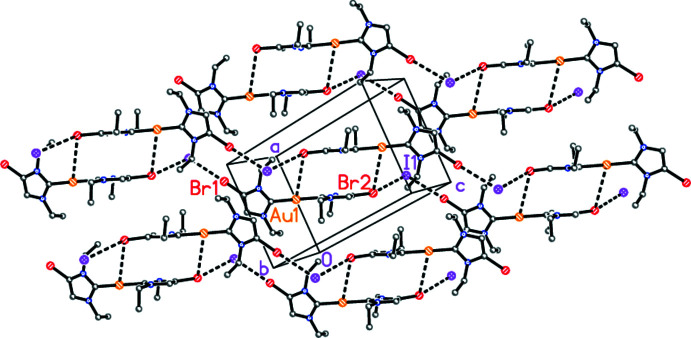
Packing diagram of compound **3** viewed perpendicular to (011). Hydrogen atoms are omitted. Dashed lines indicate halogen bonds or Au⋯Br inter­actions. Atom labels correspond to the asymmetric unit

The packing of compound **3** is shown in Fig. 4 ▸. It is dominated by short Br⋯I contacts (Table 1 ▸) that may be considered as halogen bonds (for reviews, see Metrangelo, 2008 ▸ and Cavallo *et al.*, 2016 ▸). The C—Br⋯I angles are approximately linear, whereas Br⋯I⋯Br is approximately a right angle. The anions and cations are connected to form chains with overall direction parallel to [11 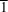 ]. The chains are in turn connected in pairs by the contact Au⋯Br2 [3.8033 (3) Å, operator 1 − *x*, 1 − *y*, 1 − *z*]. Within the double chains, the inter­centroid distance between the carbene rings based on N1 and N2 is 3.5265 (14) Å, and between the double chains the inter­centroid distance between the rings based on N3 and N4 (operator 1 − *x*, 2 − *y*, −*z*) is 3.6187 (14) Å; these offset contacts may represent π⋯π inter­actions.

**Figure 5:**
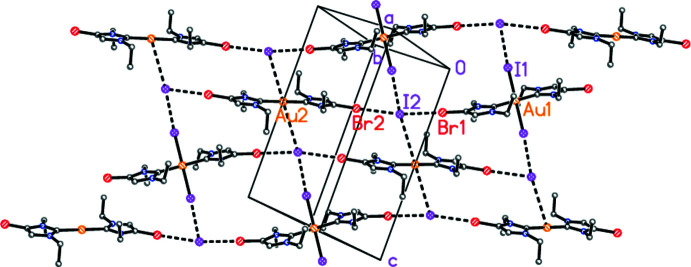
Packing diagram of compound **4** viewed perpendicular to ( 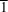 03). Hydrogen atoms are omitted. Dashed lines indicate halogen bonds or Au⋯I contacts. Atom labels correspond to the asymmetric unit.

The packing of compound **4** (Fig. 5 ▸) also involves halogen bonds. The cations are connected to form chains parallel to [331] (horizontal in Fig. 5 ▸) by contacts between each bromine atom and the iodide I2. As in **3**, the C—Br⋯I angles are approximately linear. The Au^III^ cations are further connected in the [11 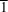 ] direction (vertical in Fig. 4 ▸) by a very short I1⋯I2 contact and a long I2⋯Au2 contact. The result is a reticular layer structure parallel to (1 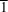 0), in which the iodide anion I2 is four-coordinate. The angle between the two chain directions is 76.4°. There are no short contacts between ring centroids.

Contact distances and angles involving the heavy atoms are included in Tables 1 ▸ and 2 ▸. Some C—H⋯Br and C—H⋯I contacts are listed in the supporting information; these might be considered as borderline hydrogen bonds.

## Database survey

Using version 2.0.5 of the CSD (Groom *et al.*, 2016 ▸), a *ConQuest* search (Bruno *et al.*, 2002 ▸) for bis­(carbene)gold(I) cations gave 355 hits, with an average Au—C bond length of 2.023 Å. For Au^III^ cations of the form [(carbene)_2_Au*X*_2_]^+^ (*X* = halogen), only 38 hits were recorded, and only six of these involved iodine as the halogen [refcodes: ANUJIE (Baron *et al.*, 2016 ▸), CIVMOK (Jothibasu *et al.*, 2008 ▸), MEZZOI (Gil-Rubio *et al.*, 2013 ▸), POYHOB (Ghosh & Catalano, 2009 ▸), XOMFIR and XONCAH (Holthoff *et al.*, 2019 ▸)]. XOMFIR presents a rare example of a non-cyclic carbene ligand. The average Au—C and Au—I bond lengths are 2.034 and 2.614 Å, respectively. The Au—C bond lengths of **3** and **4** may thus be considered normal, whereas the Au—I bond of **4** is longer than all those previously reported. It is tempting to suggest that this is associated with the halogen bonding, but MEZZOI and POYHOB also display short I⋯I contacts (3.680 and 3.478 Å, respectively), while XONCAH has a short Au⋯I contact of 3.438 Å. Short halogen⋯halogen contacts between Au^III^ species are relatively frequent; we recently drew attention to such contacts in AuCl_4_^−^ and AuBr_4_^−^ salts with protonated amine cations (Döring & Jones, 2016 ▸) but we did not include AuI_4_^−^ salts because these are far more difficult to access.

## Synthesis and crystallization

We have described the syntheses of compounds **1**, **2** (Schmidt *et al.*, 2017*b* ▸) and **3** (Schmidt *et al.*, 2017*a* ▸) elsewhere, but give a brief summary here. The reagents were purchased from Sigma—Aldrich, Alfa Aesar or TCI and used without additional purification steps. All reactions were performed without precautions to exclude air or moisture. In the first step, 4-bromo­imidazole was reacted with ethyl iodide in the presence of potassium carbonate to yield the bis­alkyl­ated imidazolium iodide (**1**) (Fig. 1 ▸). Compound **1** was then transformed in a two-step procedure by reaction with Ag_2_O and chlorido­(di­methyl­sulfide)­gold(I) to the gold(I) NHC complex **2**. The bis­carbene complex [(NHC)_2_Au]^+^ I^−^ (**3**) was obtained by further reaction of **2** with **1**.

Single crystals of complex **3** were obtained by diffusion of *n*-hexane into a solution of **3** in chloro­form/deutero­chloro­form. A few crystals of the mixed-valence complex **4** also formed, for reasons that are not clear, and the compound was identified by X-ray analysis as reported here.

## Refinement

Crystal data, data collection and structure refinement details are summarized in Table 3 ▸. For both structures, the methyl groups were refined as idealized rigid groups allowed to rotate but not tip (AFIX 137; C—H 0.98 Å, H—C—H 109.5°). The methyl­ene and NHC ring hydrogens were included using a riding model starting from calculated positions (C—H = 0.99 or 0.95 Å respectively). The *U*_iso_(H) values were fixed at 1.2 (for methyl­ene groups) or 1.5 (for methyl groups) times the *U*_eq_ value of the parent carbon atoms.

The asymmetric unit of **3** was chosen to include the short Br2⋯I1 contact. This means that the iodide lies outside the reference unit cell. Similarly, the asymmetric unit of **4** was chosen as a central I2 anion coordinated by two cations (Fig. 2 ▸). The long and narrow shape of this unit means that the centroid of the Au^III^ cation does not lie within the reference cell. In both cases, this leads to a *CheckCIF* Alert G.

The large difference peaks close to Au2 and I2 of structure **4** may be a consequence of its moderate crystal quality (somewhat irregular and diffuse reflection shapes) and/or residual absorption errors. The peaks can of course be made smaller by cutting the data to a lower 2θ_max_ value, but we prefer not to do this because the mean *I*/σ(*I*) value at highest resolution (0.74–0.71 Å) is still quite high at 8.4.

## Supplementary Material

Crystal structure: contains datablock(s) 3, 4, global. DOI: 10.1107/S2056989021011488/yz2012sup1.cif

Structure factors: contains datablock(s) 3. DOI: 10.1107/S2056989021011488/yz20123sup2.hkl

Structure factors: contains datablock(s) 4. DOI: 10.1107/S2056989021011488/yz20124sup3.hkl

CCDC references: 2119423, 2119422

Additional supporting information:  crystallographic information; 3D view; checkCIF report

## Figures and Tables

**Table 1:** Selected geometric parameters (Å, °) for **3** [Chem scheme1]

Au1—C1	2.020 (2)	I1⋯Br2	3.6072 (3)
Au1—C8	2.022 (2)	Au1⋯Br2^ii^	3.8033 (3)
I1⋯Br1^i^	3.5294 (3)		
			
C1—Au1—C8	174.97 (9)	C2—Br1⋯I1^iii^	172.43 (7)
Br1^i^⋯I1⋯Br2	101.436 (8)	C9—Br2⋯I1	162.21 (8)

Symmetry codes: (i) x-1, y-1, z+1; (ii) -x+1, -y+1, -z+1; (iii) x+1, y+1, z-1.

**Table 2:** Selected geometric parameters (Å, °) for **4** [Chem scheme1]

Au1—C1	2.033 (7)	Au2—C1′	2.018 (7)
Au1—I1	2.6564 (5)	I2⋯Br2	3.5575 (8)
I1⋯I2^i^	3.5136 (7)	I2⋯Au1^i^	4.1539 (5)
Br1⋯I2	3.4347 (8)		
			
C1^ii^—Au1—C1	180.0	Br1⋯I2⋯Br2	169.87 (2)
C1^ii^—Au1—I1	89.05 (18)	I1^i^⋯I2⋯Br2	72.852 (17)
C1—Au1—I1	90.95 (18)	Br1⋯I2⋯Au2^iv^	74.604 (16)
Au1—I1⋯I2^i^	176.29 (2)	I1^i^⋯I2⋯Au2^iv^	168.488 (16)
C2—Br1⋯I2	179.5 (2)	Br2⋯I2⋯Au2^iv^	114.871 (16)
C1′—Au2—C1′^iii^	180.0	C2′—Br2⋯I2	177.0 (2)
Br1⋯I2⋯I1^i^	97.240 (19)		

Symmetry codes: (i) -x, -y, -z; (ii) -x-1, -y-1, -z; (iii) -x+2, -y+2, -z+1; (iv) x-1, y-1, z.

**Table 3:** Experimental details

	**3**	**4**
Crystal data		
Chemical formula	[Au(C_7_H_11_BrN_2_)_2_]I	[Au(C_7_H_11_BrN_2_)_2_][AuI_2_(C_7_H_11_BrN_2_)_2_]I_2_
*M*_r_	730.04	1713.88
Crystal system, space group	Triclinic, *P*\overline{1}	Triclinic, *P*\overline{1}
Temperature (K)	100	100
*a*, *b*, *c* (Å)	8.4676 (2), 8.8248 (3), 14.0119 (5)	8.0245 (4), 8.5782 (3), 15.9814 (6)
α, β, γ (°)	76.374 (3), 85.320 (2), 85.251 (2)	91.228 (3), 96.517 (4), 92.255 (4)
*V* (Å^3^)	1011.99 (6)	1091.77 (8)
*Z*	2	1
Radiation type	Mo *K*α	Mo *K*α
μ (mm^−1^)	12.74	13.23
Crystal size (mm)	0.09 × 0.06 × 0.05	0.08 × 0.03 × 0.01

Data collection		
Diffractometer	XtaLAB Synergy, HyPix	XtaLAB Synergy, HyPix
Absorption correction	Multi-scan (*CrysAlis PRO*; Rigaku OD, 2021 ▸)	Multi-scan (*CrysAlis PRO*; Rigaku OD, 2021 ▸)
*T*_min_, *T*_max_	0.751, 1.000	0.703, 1.000
No. of measured, independent and observed [*I* > 2σ(*I*)] reflections	84327, 9374, 8185	61886, 6378, 5409
*R*_int_	0.043	0.048
(sin θ/λ)_max_ (Å^−1^)	0.840	0.704

Refinement		
*R*[*F* ^2^ > 2σ(*F* ^2^)], *wR*(*F* ^2^), *S*	0.023, 0.052, 1.02	0.039, 0.105, 1.06
No. of reflections	9374	6378
No. of parameters	203	215
H-atom treatment	H-atom parameters constrained	H-atom parameters constrained
Δρ_max_, Δρ_min_ (e Å^−3^)	1.53, −2.02	3.97, −2.66

Computer programs: *CrysAlis PRO* (Rigaku OD, 2021 ▸), *SHELXS* (Sheldrick, 2008 ▸), *SHELXL2017* (Sheldrick, 2015 ▸) and *XP* (Siemens, 1994 ▸).

## supplementary crystallographic information

### Bis(4-bromo-1,3-diethylimidazol-2-ylidene)gold(I) iodide (3) Crystal data

**Table:**

[Au(C_7_H_11_BrN_2_)_2_]I	*Z* = 2
*M**_r_* = 730.04	*F*(000) = 672
Triclinic, *P*1	*D*_x_ = 2.396 Mg m^−^^3^
*a* = 8.4676 (2) Å	Mo *K*α radiation, λ = 0.71073 Å
*b* = 8.8248 (3) Å	Cell parameters from 47541 reflections
*c* = 14.0119 (5) Å	θ = 2.7–36.4°
α = 76.374 (3)°	µ = 12.74 mm^−^^1^
β = 85.320 (2)°	*T* = 100 K
γ = 85.251 (2)°	Block, colourless
*V* = 1011.99 (6) Å^3^	0.09 × 0.06 × 0.05 mm

### Bis(4-bromo-1,3-diethylimidazol-2-ylidene)gold(I) iodide (3) Data collection

**Table:**

XtaLAB Synergy, HyPix diffractometer	9374 independent reflections
Radiation source: micro-focus sealed X-ray tube	8185 reflections with *I* > 2σ(*I*)
Detector resolution: 10.0000 pixels mm^-1^	*R*_int_ = 0.043
ω scans	θ_max_ = 36.7°, θ_min_ = 2.8°
Absorption correction: multi-scan (CrysAlisPro; Rigaku OD, 2021)	*h* = −14→14
*T*_min_ = 0.751, *T*_max_ = 1.000	*k* = −14→14
84327 measured reflections	*l* = −22→22

### Bis(4-bromo-1,3-diethylimidazol-2-ylidene)gold(I) iodide (3) Refinement

**Table:**

Refinement on *F*^2^	0 restraints
Least-squares matrix: full	Hydrogen site location: inferred from neighbouring sites
*R*[*F*^2^ > 2σ(*F*^2^)] = 0.023	H-atom parameters constrained
*wR*(*F*^2^) = 0.052	*w* = 1/[σ^2^(*F*_o_^2^) + (0.0219*P*)^2^ + 1.6441*P*] where *P* = (*F*_o_^2^ + 2*F*_c_^2^)/3
*S* = 1.02	(Δ/σ)_max_ = 0.002
9374 reflections	Δρ_max_ = 1.53 e Å^−^^3^
203 parameters	Δρ_min_ = −2.02 e Å^−^^3^

### Bis(4-bromo-1,3-diethylimidazol-2-ylidene)gold(I) iodide (3) Special details

**Table:**

Geometry. All esds (except the esd in the dihedral angle between two l.s. planes) are estimated using the full covariance matrix. The cell esds are taken into account individually in the estimation of esds in distances, angles and torsion angles; correlations between esds in cell parameters are only used when they are defined by crystal symmetry. An approximate (isotropic) treatment of cell esds is used for estimating esds involving l.s. planes.

### Bis(4-bromo-1,3-diethylimidazol-2-ylidene)gold(I) iodide (3) Fractional atomic coordinates and isotropic or equivalent isotropic displacement parameters (Å^2^)

**Table:**

	*x*	*y*	*z*	*U*_iso_*/*U*_eq_	
Au1	0.46224 (2)	0.71713 (2)	0.23616 (2)	0.01505 (2)	
I1	0.25568 (2)	−0.12579 (2)	0.72649 (2)	0.01947 (3)	
Br1	0.90742 (3)	0.85910 (3)	−0.11585 (2)	0.01837 (4)	
Br2	0.22321 (3)	0.27369 (3)	0.58678 (2)	0.02294 (5)	
N1	0.7026 (2)	0.8013 (2)	0.06168 (14)	0.0144 (3)	
N2	0.4687 (2)	0.7981 (2)	0.01341 (14)	0.0151 (3)	
N3	0.3414 (2)	0.4840 (2)	0.41402 (14)	0.0158 (3)	
N4	0.3267 (2)	0.7110 (2)	0.44721 (14)	0.0168 (3)	
C1	0.5490 (3)	0.7807 (3)	0.09487 (17)	0.0152 (4)	
C2	0.7156 (3)	0.8303 (3)	−0.03991 (16)	0.0150 (4)	
C3	0.5681 (3)	0.8289 (3)	−0.07101 (17)	0.0159 (4)	
H3	0.539432	0.845625	−0.137005	0.019*	
C4	0.8329 (3)	0.7876 (3)	0.12682 (17)	0.0190 (4)	
H4A	0.796328	0.835665	0.182530	0.023*	
H4B	0.922392	0.845773	0.090037	0.023*	
C5	0.8904 (3)	0.6186 (3)	0.1667 (2)	0.0268 (5)	
H5A	0.802277	0.560658	0.203377	0.040*	
H5B	0.975985	0.614518	0.210455	0.040*	
H5C	0.930249	0.571616	0.111832	0.040*	
C6	0.3003 (3)	0.7693 (3)	0.01381 (19)	0.0190 (4)	
H6A	0.251033	0.845711	−0.041004	0.023*	
H6B	0.244648	0.783981	0.076250	0.023*	
C7	0.2815 (3)	0.6044 (3)	0.0029 (2)	0.0253 (5)	
H7A	0.335813	0.590123	−0.059152	0.038*	
H7B	0.168425	0.588003	0.002906	0.038*	
H7C	0.328132	0.528699	0.057982	0.038*	
C8	0.3723 (3)	0.6350 (3)	0.37528 (17)	0.0164 (4)	
C9	0.2739 (3)	0.4672 (3)	0.50878 (17)	0.0175 (4)	
C10	0.2644 (3)	0.6105 (3)	0.53014 (17)	0.0190 (4)	
H10	0.223233	0.636119	0.589902	0.023*	
C11	0.3707 (3)	0.3610 (3)	0.35936 (19)	0.0208 (4)	
H11A	0.380137	0.258081	0.406376	0.025*	
H11B	0.472452	0.376079	0.319289	0.025*	
C12	0.2385 (4)	0.3624 (3)	0.2928 (2)	0.0292 (6)	
H12A	0.137555	0.348228	0.332223	0.044*	
H12B	0.260413	0.277190	0.258600	0.044*	
H12C	0.231993	0.462511	0.244325	0.044*	
C13	0.3265 (3)	0.8808 (3)	0.43601 (19)	0.0204 (4)	
H13A	0.407523	0.923764	0.383801	0.024*	
H13B	0.354381	0.903198	0.498325	0.024*	
C14	0.1657 (4)	0.9582 (3)	0.4096 (3)	0.0367 (7)	
H14A	0.140898	0.940989	0.346040	0.055*	
H14B	0.166327	1.070607	0.405280	0.055*	
H14C	0.085156	0.913349	0.460390	0.055*	

### Bis(4-bromo-1,3-diethylimidazol-2-ylidene)gold(I) iodide (3) Atomic displacement parameters (Å^2^)

**Table:**

	*U*^11^	*U*^22^	*U*^33^	*U*^12^	*U*^13^	*U*^23^
Au1	0.01531 (4)	0.01589 (4)	0.01371 (4)	−0.00177 (3)	0.00196 (3)	−0.00363 (3)
I1	0.02119 (7)	0.02104 (7)	0.01555 (6)	0.00120 (5)	0.00096 (5)	−0.00454 (5)
Br1	0.01523 (10)	0.02506 (11)	0.01603 (9)	−0.00645 (8)	0.00254 (7)	−0.00658 (8)
Br2	0.03080 (13)	0.02037 (11)	0.01701 (10)	−0.00987 (9)	−0.00213 (9)	−0.00004 (8)
N1	0.0136 (8)	0.0164 (8)	0.0137 (8)	−0.0021 (6)	0.0003 (6)	−0.0045 (6)
N2	0.0129 (8)	0.0163 (8)	0.0155 (8)	−0.0007 (6)	0.0000 (6)	−0.0031 (7)
N3	0.0170 (8)	0.0159 (8)	0.0149 (8)	−0.0017 (7)	−0.0007 (6)	−0.0040 (7)
N4	0.0204 (9)	0.0153 (8)	0.0148 (8)	−0.0032 (7)	−0.0003 (7)	−0.0035 (7)
C1	0.0139 (9)	0.0155 (9)	0.0165 (9)	−0.0023 (7)	0.0017 (7)	−0.0046 (7)
C2	0.0152 (9)	0.0172 (9)	0.0128 (8)	−0.0035 (7)	0.0011 (7)	−0.0038 (7)
C3	0.0149 (9)	0.0178 (10)	0.0153 (9)	−0.0021 (7)	0.0001 (7)	−0.0045 (8)
C4	0.0176 (10)	0.0256 (11)	0.0157 (9)	−0.0056 (8)	−0.0020 (8)	−0.0070 (8)
C5	0.0241 (12)	0.0309 (14)	0.0253 (12)	0.0050 (10)	−0.0074 (10)	−0.0065 (10)
C6	0.0119 (9)	0.0231 (11)	0.0217 (10)	−0.0001 (8)	−0.0017 (8)	−0.0049 (9)
C7	0.0201 (11)	0.0243 (12)	0.0330 (13)	−0.0066 (9)	−0.0003 (10)	−0.0081 (10)
C8	0.0153 (9)	0.0167 (9)	0.0169 (9)	−0.0016 (7)	−0.0004 (7)	−0.0036 (8)
C9	0.0211 (10)	0.0171 (10)	0.0136 (9)	−0.0045 (8)	−0.0012 (8)	−0.0011 (8)
C10	0.0218 (11)	0.0199 (10)	0.0151 (9)	−0.0047 (8)	0.0014 (8)	−0.0037 (8)
C11	0.0251 (12)	0.0174 (10)	0.0212 (11)	−0.0008 (9)	−0.0005 (9)	−0.0075 (9)
C12	0.0391 (16)	0.0252 (13)	0.0264 (13)	−0.0052 (11)	−0.0088 (11)	−0.0085 (10)
C13	0.0271 (12)	0.0143 (10)	0.0200 (10)	−0.0054 (8)	0.0024 (9)	−0.0042 (8)
C14	0.0328 (16)	0.0179 (12)	0.056 (2)	0.0019 (11)	−0.0004 (14)	−0.0034 (13)

### Bis(4-bromo-1,3-diethylimidazol-2-ylidene)gold(I) iodide (3) Geometric parameters (Å, º)

**Table:**

Au1—C1	2.020 (2)	C13—C14	1.506 (4)
Au1—C8	2.022 (2)	C3—H3	0.9500
I1—Br1^i^	3.5294 (3)	C4—H4A	0.9900
I1—Br2	3.6072 (3)	C4—H4B	0.9900
Au1—Br2^ii^	3.8033 (3)	C5—H5A	0.9800
Br1—C2	1.869 (2)	C5—H5B	0.9800
Br2—C9	1.861 (2)	C5—H5C	0.9800
N1—C1	1.357 (3)	C6—H6A	0.9900
N1—C2	1.382 (3)	C6—H6B	0.9900
N1—C4	1.468 (3)	C7—H7A	0.9800
N2—C1	1.348 (3)	C7—H7B	0.9800
N2—C3	1.381 (3)	C7—H7C	0.9800
N2—C6	1.468 (3)	C10—H10	0.9500
N3—C8	1.354 (3)	C11—H11A	0.9900
N3—C9	1.382 (3)	C11—H11B	0.9900
N3—C11	1.464 (3)	C12—H12A	0.9800
N4—C8	1.351 (3)	C12—H12B	0.9800
N4—C10	1.381 (3)	C12—H12C	0.9800
N4—C13	1.469 (3)	C13—H13A	0.9900
C2—C3	1.357 (3)	C13—H13B	0.9900
C4—C5	1.518 (4)	C14—H14A	0.9800
C6—C7	1.521 (4)	C14—H14B	0.9800
C9—C10	1.361 (3)	C14—H14C	0.9800
C11—C12	1.512 (4)		
			
C1—Au1—C8	174.97 (9)	C4—C5—H5A	109.5
Br1^i^—I1—Br2	101.436 (8)	C4—C5—H5B	109.5
C2—Br1—I1^iii^	172.43 (7)	H5A—C5—H5B	109.5
C9—Br2—I1	162.21 (8)	C4—C5—H5C	109.5
C1—N1—C2	109.82 (19)	H5A—C5—H5C	109.5
C1—N1—C4	123.48 (19)	H5B—C5—H5C	109.5
C2—N1—C4	126.65 (19)	N2—C6—H6A	109.5
C1—N2—C3	111.61 (19)	C7—C6—H6A	109.5
C1—N2—C6	124.54 (19)	N2—C6—H6B	109.5
C3—N2—C6	123.46 (19)	C7—C6—H6B	109.5
C8—N3—C9	110.15 (19)	H6A—C6—H6B	108.1
C8—N3—C11	123.4 (2)	C6—C7—H7A	109.5
C9—N3—C11	126.4 (2)	C6—C7—H7B	109.5
C8—N4—C10	111.1 (2)	H7A—C7—H7B	109.5
C8—N4—C13	124.9 (2)	C6—C7—H7C	109.5
C10—N4—C13	123.7 (2)	H7A—C7—H7C	109.5
N2—C1—N1	105.25 (19)	H7B—C7—H7C	109.5
N2—C1—Au1	127.11 (16)	C9—C10—H10	127.0
N1—C1—Au1	127.41 (17)	N4—C10—H10	127.0
C3—C2—N1	107.77 (19)	N3—C11—H11A	109.3
C3—C2—Br1	128.16 (17)	C12—C11—H11A	109.3
N1—C2—Br1	124.05 (17)	N3—C11—H11B	109.3
C2—C3—N2	105.6 (2)	C12—C11—H11B	109.3
N1—C4—C5	112.1 (2)	H11A—C11—H11B	108.0
N2—C6—C7	110.9 (2)	C11—C12—H12A	109.5
N4—C8—N3	105.41 (19)	C11—C12—H12B	109.5
N4—C8—Au1	130.27 (17)	H12A—C12—H12B	109.5
N3—C8—Au1	124.30 (17)	C11—C12—H12C	109.5
C10—C9—N3	107.3 (2)	H12A—C12—H12C	109.5
C10—C9—Br2	130.48 (18)	H12B—C12—H12C	109.5
N3—C9—Br2	122.09 (17)	N4—C13—H13A	109.5
C9—C10—N4	106.0 (2)	C14—C13—H13A	109.5
N3—C11—C12	111.5 (2)	N4—C13—H13B	109.5
N4—C13—C14	110.6 (2)	C14—C13—H13B	109.5
C2—C3—H3	127.2	H13A—C13—H13B	108.1
N2—C3—H3	127.2	C13—C14—H14A	109.5
N1—C4—H4A	109.2	C13—C14—H14B	109.5
C5—C4—H4A	109.2	H14A—C14—H14B	109.5
N1—C4—H4B	109.2	C13—C14—H14C	109.5
C5—C4—H4B	109.2	H14A—C14—H14C	109.5
H4A—C4—H4B	107.9	H14B—C14—H14C	109.5
			
C3—N2—C1—N1	0.2 (3)	C13—N4—C8—N3	−175.0 (2)
C6—N2—C1—N1	173.2 (2)	C10—N4—C8—Au1	177.04 (18)
C3—N2—C1—Au1	−174.55 (17)	C13—N4—C8—Au1	3.3 (4)
C6—N2—C1—Au1	−1.5 (3)	C9—N3—C8—N4	1.3 (3)
C2—N1—C1—N2	−0.3 (2)	C11—N3—C8—N4	178.8 (2)
C4—N1—C1—N2	−178.1 (2)	C9—N3—C8—Au1	−177.17 (17)
C2—N1—C1—Au1	174.36 (16)	C11—N3—C8—Au1	0.4 (3)
C4—N1—C1—Au1	−3.4 (3)	C8—N3—C9—C10	−0.8 (3)
C1—N1—C2—C3	0.4 (3)	C11—N3—C9—C10	−178.3 (2)
C4—N1—C2—C3	178.1 (2)	C8—N3—C9—Br2	−177.42 (17)
C1—N1—C2—Br1	−177.92 (17)	C11—N3—C9—Br2	5.1 (3)
C4—N1—C2—Br1	−0.2 (3)	I1—Br2—C9—C10	−114.8 (3)
N1—C2—C3—N2	−0.3 (3)	I1—Br2—C9—N3	61.0 (4)
Br1—C2—C3—N2	177.95 (17)	N3—C9—C10—N4	0.0 (3)
C1—N2—C3—C2	0.1 (3)	Br2—C9—C10—N4	176.24 (19)
C6—N2—C3—C2	−173.1 (2)	C8—N4—C10—C9	0.8 (3)
C1—N1—C4—C5	81.0 (3)	C13—N4—C10—C9	174.6 (2)
C2—N1—C4—C5	−96.4 (3)	C8—N3—C11—C12	−80.8 (3)
C1—N2—C6—C7	−94.9 (3)	C9—N3—C11—C12	96.4 (3)
C3—N2—C6—C7	77.4 (3)	C8—N4—C13—C14	94.0 (3)
C10—N4—C8—N3	−1.3 (3)	C10—N4—C13—C14	−79.0 (3)

Symmetry codes: (i) *x*−1, *y*−1, *z*+1; (ii) −*x*+1, −*y*+1, −*z*+1; (iii) *x*+1, *y*+1, *z*−1.

### Bis(4-bromo-1,3-diethylimidazol-2-ylidene)gold(I) iodide (3) Hydrogen-bond geometry (Å, º)

**Table:**

*D*—H···*A*	*D*—H	H···*A*	*D*···*A*	*D*—H···*A*
C3—H3···I1^iv^	0.95	3.15	3.966 (2)	145
C4—H4*A*···I1^ii^	0.99	3.10	3.995 (3)	151
C6—H6*A*···I1^iv^	0.99	3.21	3.955 (3)	134
C10—H10···I1^v^	0.95	3.20	3.993 (2)	142
C11—H11*A*···Br2	0.99	2.79	3.265 (3)	110
C11—H11*B*···I1^vi^	0.99	3.19	3.903 (3)	130
C12—H12*B*···Br1^vii^	0.98	3.06	3.831 (3)	137
C13—H13*B*···I1^v^	0.99	3.19	4.053 (3)	146

Symmetry codes: (ii) −*x*+1, −*y*+1, −*z*+1; (iv) *x*, *y*+1, *z*−1; (v) *x*, *y*+1, *z*; (vi) −*x*+1, −*y*, −*z*+1; (vii) −*x*+1, −*y*+1, −*z*.

### Bis(4-bromo-1,3-diethylimidazol-2-ylidene)gold(I) trans-bis(4-bromo-1,3-diethyl-imidazol-2-ylidene)diiodidogold(III) diiodide (4) Crystal data

**Table:**

[Au(C_7_H_11_BrN_2_)_2_][AuI_2_(C_7_H_11_BrN_2_)_2_]I_2_	*Z* = 1
*M**_r_* = 1713.88	*F*(000) = 778
Triclinic, *P*1	*D*_x_ = 2.607 Mg m^−^^3^
*a* = 8.0245 (4) Å	Mo *K*α radiation, λ = 0.71073 Å
*b* = 8.5782 (3) Å	Cell parameters from 24546 reflections
*c* = 15.9814 (6) Å	θ = 2.6–34.1°
α = 91.228 (3)°	µ = 13.23 mm^−^^1^
β = 96.517 (4)°	*T* = 100 K
γ = 92.255 (4)°	Plate, brown
*V* = 1091.77 (8) Å^3^	0.08 × 0.03 × 0.01 mm

### Bis(4-bromo-1,3-diethylimidazol-2-ylidene)gold(I) trans-bis(4-bromo-1,3-diethyl-imidazol-2-ylidene)diiodidogold(III) diiodide (4) Data collection

**Table:**

XtaLAB Synergy, HyPix diffractometer	6378 independent reflections
Radiation source: micro-focus sealed X-ray tube	5409 reflections with *I* > 2σ(*I*)
Detector resolution: 10.0000 pixels mm^-1^	*R*_int_ = 0.048
ω scans	θ_max_ = 30.0°, θ_min_ = 2.7°
Absorption correction: multi-scan (CrysAlisPro; Rigaku OD, 2021)	*h* = −11→11
*T*_min_ = 0.703, *T*_max_ = 1.000	*k* = −12→12
61886 measured reflections	*l* = −22→22

### Bis(4-bromo-1,3-diethylimidazol-2-ylidene)gold(I) trans-bis(4-bromo-1,3-diethyl-imidazol-2-ylidene)diiodidogold(III) diiodide (4) Refinement

**Table:**

Refinement on *F*^2^	0 restraints
Least-squares matrix: full	Hydrogen site location: inferred from neighbouring sites
*R*[*F*^2^ > 2σ(*F*^2^)] = 0.039	H-atom parameters constrained
*wR*(*F*^2^) = 0.105	*w* = 1/[σ^2^(*F*_o_^2^) + (0.0494*P*)^2^ + 12.4607*P*] where *P* = (*F*_o_^2^ + 2*F*_c_^2^)/3
*S* = 1.06	(Δ/σ)_max_ < 0.001
6378 reflections	Δρ_max_ = 3.97 e Å^−^^3^
215 parameters	Δρ_min_ = −2.66 e Å^−^^3^

### Bis(4-bromo-1,3-diethylimidazol-2-ylidene)gold(I) trans-bis(4-bromo-1,3-diethyl-imidazol-2-ylidene)diiodidogold(III) diiodide (4) Special details

**Table:**

Geometry. All esds (except the esd in the dihedral angle between two l.s. planes) are estimated using the full covariance matrix. The cell esds are taken into account individually in the estimation of esds in distances, angles and torsion angles; correlations between esds in cell parameters are only used when they are defined by crystal symmetry. An approximate (isotropic) treatment of cell esds is used for estimating esds involving l.s. planes.

### Bis(4-bromo-1,3-diethylimidazol-2-ylidene)gold(I) trans-bis(4-bromo-1,3-diethyl-imidazol-2-ylidene)diiodidogold(III) diiodide (4) Fractional atomic coordinates and isotropic or equivalent isotropic displacement parameters (Å^2^)

**Table:**

	*x*	*y*	*z*	*U*_iso_*/*U*_eq_	
Au1	−0.500000	−0.500000	0.000000	0.01890 (8)	
I1	−0.36292 (6)	−0.37952 (6)	−0.12916 (3)	0.02666 (11)	
Br1	−0.00401 (10)	−0.09434 (9)	0.21316 (5)	0.02965 (16)	
N1	−0.2548 (7)	−0.2807 (7)	0.1082 (4)	0.0227 (11)	
N2	−0.1732 (7)	−0.5172 (7)	0.1164 (4)	0.0218 (11)	
C1	−0.2957 (9)	−0.4283 (8)	0.0806 (4)	0.0199 (12)	
C2	−0.1053 (9)	−0.2781 (8)	0.1624 (4)	0.0236 (13)	
C3	−0.0556 (9)	−0.4261 (8)	0.1680 (4)	0.0242 (13)	
H3	0.041194	−0.461233	0.200890	0.029*	
C4	−0.3602 (10)	−0.1472 (8)	0.0908 (5)	0.0282 (15)	
H4A	−0.426098	−0.163377	0.034872	0.034*	
H4B	−0.287389	−0.051785	0.088776	0.034*	
C5	−0.4806 (13)	−0.1229 (11)	0.1572 (6)	0.042 (2)	
H5A	−0.548069	−0.219305	0.162128	0.064*	
H5B	−0.554816	−0.038287	0.140329	0.064*	
H5C	−0.415991	−0.095597	0.211660	0.064*	
C6	−0.1643 (9)	−0.6878 (8)	0.1038 (4)	0.0243 (13)	
H6A	−0.276204	−0.738050	0.107783	0.029*	
H6B	−0.084560	−0.728786	0.149017	0.029*	
C7	−0.1087 (11)	−0.7291 (10)	0.0196 (5)	0.0343 (17)	
H7A	−0.194248	−0.699822	−0.025403	0.051*	
H7B	−0.093445	−0.841752	0.015793	0.051*	
H7C	−0.002321	−0.672810	0.013535	0.051*	
Au2	1.000000	1.000000	0.500000	0.02101 (9)	
I2	0.18002 (6)	0.24488 (5)	0.30519 (3)	0.02377 (10)	
Br2	0.40896 (9)	0.59948 (8)	0.36840 (4)	0.02459 (14)	
N1'	0.7026 (7)	0.7839 (6)	0.4369 (3)	0.0198 (11)	
N2'	0.6387 (8)	1.0226 (6)	0.4186 (4)	0.0219 (11)	
C1'	0.7658 (9)	0.9331 (7)	0.4491 (4)	0.0203 (12)	
C2'	0.5388 (9)	0.7821 (8)	0.3989 (4)	0.0216 (12)	
C3'	0.4977 (9)	0.9322 (8)	0.3871 (4)	0.0215 (12)	
H3'	0.393497	0.968182	0.362203	0.026*	
C4'	0.8021 (9)	0.6466 (8)	0.4567 (4)	0.0224 (13)	
H4'1	0.725634	0.555349	0.463211	0.027*	
H4'2	0.873672	0.665833	0.510905	0.027*	
C5'	0.9122 (10)	0.6105 (8)	0.3881 (5)	0.0290 (15)	
H5'1	0.841801	0.593654	0.334152	0.044*	
H5'2	0.973515	0.516180	0.402016	0.044*	
H5'3	0.992338	0.698354	0.383758	0.044*	
C6'	0.6523 (10)	1.1916 (8)	0.4105 (5)	0.0259 (14)	
H6'1	0.746773	1.235149	0.450502	0.031*	
H6'2	0.547969	1.238037	0.425063	0.031*	
C7'	0.6810 (9)	1.2344 (8)	0.3214 (5)	0.0281 (15)	
H7'1	0.785274	1.189945	0.307254	0.042*	
H7'2	0.689663	1.348279	0.317476	0.042*	
H7'3	0.586704	1.192742	0.281833	0.042*	

### Bis(4-bromo-1,3-diethylimidazol-2-ylidene)gold(I) trans-bis(4-bromo-1,3-diethyl-imidazol-2-ylidene)diiodidogold(III) diiodide (4) Atomic displacement parameters (Å^2^)

**Table:**

	*U*^11^	*U*^22^	*U*^33^	*U*^12^	*U*^13^	*U*^23^
Au1	0.02247 (17)	0.01893 (16)	0.01489 (15)	−0.00122 (12)	0.00110 (12)	0.00024 (12)
I1	0.0288 (2)	0.0301 (2)	0.0214 (2)	−0.00043 (18)	0.00418 (16)	0.00327 (17)
Br1	0.0348 (4)	0.0248 (3)	0.0272 (3)	−0.0053 (3)	−0.0026 (3)	−0.0047 (3)
N1	0.025 (3)	0.023 (3)	0.020 (3)	0.000 (2)	0.004 (2)	−0.002 (2)
N2	0.022 (3)	0.021 (3)	0.021 (3)	−0.002 (2)	−0.001 (2)	−0.003 (2)
C1	0.028 (3)	0.018 (3)	0.013 (3)	−0.002 (2)	0.002 (2)	−0.001 (2)
C2	0.028 (3)	0.024 (3)	0.018 (3)	−0.004 (3)	0.001 (2)	−0.003 (2)
C3	0.028 (3)	0.027 (3)	0.017 (3)	0.000 (3)	0.000 (2)	−0.004 (2)
C4	0.039 (4)	0.019 (3)	0.025 (3)	−0.001 (3)	0.000 (3)	−0.002 (3)
C5	0.051 (5)	0.035 (4)	0.042 (5)	0.004 (4)	0.014 (4)	−0.008 (4)
C6	0.028 (3)	0.021 (3)	0.024 (3)	0.001 (3)	0.002 (3)	0.000 (3)
C7	0.042 (5)	0.031 (4)	0.031 (4)	0.005 (3)	0.005 (3)	−0.007 (3)
Au2	0.02690 (18)	0.01581 (16)	0.01892 (16)	0.00103 (12)	−0.00311 (13)	−0.00180 (12)
I2	0.0231 (2)	0.0219 (2)	0.0252 (2)	−0.00092 (15)	−0.00093 (16)	−0.00113 (16)
Br2	0.0266 (3)	0.0199 (3)	0.0267 (3)	−0.0050 (2)	0.0032 (3)	−0.0015 (2)
N1'	0.028 (3)	0.014 (2)	0.017 (2)	−0.001 (2)	0.001 (2)	0.0007 (19)
N2'	0.028 (3)	0.015 (2)	0.022 (3)	0.001 (2)	−0.001 (2)	−0.002 (2)
C1'	0.031 (3)	0.014 (3)	0.015 (3)	0.004 (2)	0.000 (2)	0.000 (2)
C2'	0.024 (3)	0.020 (3)	0.022 (3)	0.000 (2)	0.005 (2)	0.000 (2)
C3'	0.025 (3)	0.017 (3)	0.021 (3)	0.000 (2)	−0.002 (2)	−0.002 (2)
C4'	0.026 (3)	0.016 (3)	0.026 (3)	0.007 (2)	0.008 (3)	0.002 (2)
C5'	0.031 (4)	0.019 (3)	0.039 (4)	0.008 (3)	0.010 (3)	0.001 (3)
C6'	0.031 (4)	0.015 (3)	0.029 (3)	0.002 (3)	−0.005 (3)	−0.001 (3)
C7'	0.025 (3)	0.022 (3)	0.038 (4)	0.000 (3)	0.008 (3)	0.008 (3)

### Bis(4-bromo-1,3-diethylimidazol-2-ylidene)gold(I) trans-bis(4-bromo-1,3-diethyl-imidazol-2-ylidene)diiodidogold(III) diiodide (4) Geometric parameters (Å, º)

**Table:**

Au1—C1^i^	2.033 (7)	N2'—C6'	1.459 (9)
Au1—C1	2.033 (7)	C2'—C3'	1.353 (9)
Au1—I1^i^	2.6564 (5)	C4'—C5'	1.519 (10)
Au1—I1	2.6564 (5)	C6'—C7'	1.519 (11)
I1—I2^ii^	3.5136 (7)	C3—H3	0.9500
Br1—C2	1.870 (7)	C4—H4A	0.9900
Br1—I2	3.4347 (8)	C4—H4B	0.9900
N1—C1	1.347 (8)	C5—H5A	0.9800
N1—C2	1.397 (9)	C5—H5B	0.9800
N1—C4	1.464 (10)	C5—H5C	0.9800
N2—C1	1.351 (9)	C6—H6A	0.9900
N2—C3	1.385 (8)	C6—H6B	0.9900
N2—C6	1.479 (9)	C7—H7A	0.9800
C2—C3	1.348 (10)	C7—H7B	0.9800
C4—C5	1.530 (12)	C7—H7C	0.9800
C6—C7	1.504 (11)	C3'—H3'	0.9500
Au2—C1'	2.018 (7)	C4'—H4'1	0.9900
Au2—C1'^iii^	2.018 (7)	C4'—H4'2	0.9900
I2—Br2	3.5575 (8)	C5'—H5'1	0.9800
I2—Au1^ii^	4.1539 (5)	C5'—H5'2	0.9800
Br2—C2'	1.871 (7)	C5'—H5'3	0.9800
N1'—C1'	1.360 (8)	C6'—H6'1	0.9900
N1'—C2'	1.383 (9)	C6'—H6'2	0.9900
N1'—C4'	1.469 (8)	C7'—H7'1	0.9800
N2'—C1'	1.354 (9)	C7'—H7'2	0.9800
N2'—C3'	1.386 (9)	C7'—H7'3	0.9800
			
C1^i^—Au1—C1	180.0	N2—C3—H3	126.7
C1^i^—Au1—I1^i^	90.95 (18)	N1—C4—H4A	109.1
C1—Au1—I1^i^	89.05 (18)	C5—C4—H4A	109.1
C1^i^—Au1—I1	89.05 (18)	N1—C4—H4B	109.1
C1—Au1—I1	90.95 (18)	C5—C4—H4B	109.1
I1^i^—Au1—I1	180.0	H4A—C4—H4B	107.8
Au1—I1—I2^ii^	176.29 (2)	C4—C5—H5A	109.5
C2—Br1—I2	179.5 (2)	C4—C5—H5B	109.5
C1—N1—C2	109.5 (6)	H5A—C5—H5B	109.5
C1—N1—C4	124.8 (6)	C4—C5—H5C	109.5
C2—N1—C4	125.3 (6)	H5A—C5—H5C	109.5
C1—N2—C3	110.4 (6)	H5B—C5—H5C	109.5
C1—N2—C6	125.6 (6)	N2—C6—H6A	109.3
C3—N2—C6	124.0 (6)	C7—C6—H6A	109.3
N1—C1—N2	106.2 (6)	N2—C6—H6B	109.3
N1—C1—Au1	126.4 (5)	C7—C6—H6B	109.3
N2—C1—Au1	127.4 (5)	H6A—C6—H6B	107.9
C3—C2—N1	107.2 (6)	C6—C7—H7A	109.5
C3—C2—Br1	129.9 (6)	C6—C7—H7B	109.5
N1—C2—Br1	122.9 (5)	H7A—C7—H7B	109.5
C2—C3—N2	106.6 (6)	C6—C7—H7C	109.5
N1—C4—C5	112.6 (7)	H7A—C7—H7C	109.5
N2—C6—C7	111.8 (6)	H7B—C7—H7C	109.5
C1'—Au2—C1'^iii^	180.0	C2'—C3'—H3'	127.0
Br1—I2—I1^ii^	97.240 (19)	N2'—C3'—H3'	127.0
Br1—I2—Br2	169.87 (2)	N1'—C4'—H4'1	109.3
I1^ii^—I2—Br2	72.852 (17)	C5'—C4'—H4'1	109.3
Br1—I2—Au2^iv^	74.604 (16)	N1'—C4'—H4'2	109.3
I1^ii^—I2—Au2^iv^	168.488 (16)	C5'—C4'—H4'2	109.3
Br2—I2—Au2^iv^	114.871 (16)	H4'1—C4'—H4'2	108.0
C2'—Br2—I2	177.0 (2)	C4'—C5'—H5'1	109.5
C1'—N1'—C2'	110.4 (6)	C4'—C5'—H5'2	109.5
C1'—N1'—C4'	123.3 (6)	H5'1—C5'—H5'2	109.5
C2'—N1'—C4'	126.1 (6)	C4'—C5'—H5'3	109.5
C1'—N2'—C3'	111.4 (6)	H5'1—C5'—H5'3	109.5
C1'—N2'—C6'	124.9 (6)	H5'2—C5'—H5'3	109.5
C3'—N2'—C6'	123.3 (6)	N2'—C6'—H6'1	109.4
N2'—C1'—N1'	104.7 (6)	C7'—C6'—H6'1	109.4
N2'—C1'—Au2	128.9 (5)	N2'—C6'—H6'2	109.4
N1'—C1'—Au2	126.4 (5)	C7'—C6'—H6'2	109.4
C3'—C2'—N1'	107.4 (6)	H6'1—C6'—H6'2	108.0
C3'—C2'—Br2	128.7 (5)	C6'—C7'—H7'1	109.5
N1'—C2'—Br2	123.9 (5)	C6'—C7'—H7'2	109.5
C2'—C3'—N2'	105.9 (6)	H7'1—C7'—H7'2	109.5
N1'—C4'—C5'	111.4 (6)	C6'—C7'—H7'3	109.5
N2'—C6'—C7'	111.0 (6)	H7'1—C7'—H7'3	109.5
C2—C3—H3	126.7	H7'2—C7'—H7'3	109.5
			
C2—N1—C1—N2	−0.4 (7)	C3'—N2'—C1'—N1'	−0.4 (8)
C4—N1—C1—N2	−174.5 (6)	C6'—N2'—C1'—N1'	−173.8 (6)
C2—N1—C1—Au1	−179.8 (5)	C3'—N2'—C1'—Au2	178.6 (5)
C4—N1—C1—Au1	6.2 (10)	C6'—N2'—C1'—Au2	5.3 (10)
C3—N2—C1—N1	1.0 (8)	C2'—N1'—C1'—N2'	0.4 (7)
C6—N2—C1—N1	−179.5 (6)	C4'—N1'—C1'—N2'	176.4 (6)
C3—N2—C1—Au1	−179.7 (5)	C2'—N1'—C1'—Au2	−178.8 (5)
C6—N2—C1—Au1	−0.2 (10)	C4'—N1'—C1'—Au2	−2.7 (9)
C1—N1—C2—C3	−0.3 (8)	C1'—N1'—C2'—C3'	−0.1 (8)
C4—N1—C2—C3	173.8 (7)	C4'—N1'—C2'—C3'	−176.1 (6)
C1—N1—C2—Br1	179.1 (5)	C1'—N1'—C2'—Br2	178.1 (5)
C4—N1—C2—Br1	−6.8 (10)	C4'—N1'—C2'—Br2	2.2 (9)
N1—C2—C3—N2	0.9 (8)	N1'—C2'—C3'—N2'	−0.1 (8)
Br1—C2—C3—N2	−178.5 (5)	Br2—C2'—C3'—N2'	−178.3 (5)
C1—N2—C3—C2	−1.2 (8)	C1'—N2'—C3'—C2'	0.4 (8)
C6—N2—C3—C2	179.3 (6)	C6'—N2'—C3'—C2'	173.8 (6)
C1—N1—C4—C5	89.5 (8)	C1'—N1'—C4'—C5'	−79.6 (8)
C2—N1—C4—C5	−83.7 (9)	C2'—N1'—C4'—C5'	95.8 (8)
C1—N2—C6—C7	76.1 (9)	C1'—N2'—C6'—C7'	97.4 (8)
C3—N2—C6—C7	−104.5 (8)	C3'—N2'—C6'—C7'	−75.2 (9)

Symmetry codes: (i) −*x*−1, −*y*−1, −*z*; (ii) −*x*, −*y*, −*z*; (iii) −*x*+2, −*y*+2, −*z*+1; (iv) *x*−1, *y*−1, *z*.

### Bis(4-bromo-1,3-diethylimidazol-2-ylidene)gold(I) trans-bis(4-bromo-1,3-diethyl-imidazol-2-ylidene)diiodidogold(III) diiodide (4) Hydrogen-bond geometry (Å, º)

**Table:**

*D*—H···*A*	*D*—H	H···*A*	*D*···*A*	*D*—H···*A*
C3—H3···I1^v^	0.95	3.28	3.913 (7)	126
C3—H3···I2^vi^	0.95	3.22	4.011 (7)	142
C4—H4*A*···I1	0.99	3.28	4.002 (7)	132
C6—H6*A*···I1^i^	0.99	3.16	3.925 (7)	136
C6—H6*B*···I2^vi^	0.99	3.11	4.064 (7)	163
C7—H7*C*···I1^v^	0.98	3.29	4.051 (9)	136
C3′—H3′···I2^vii^	0.95	3.08	3.916 (7)	148
C4′—H4′2···I2^viii^	0.99	3.10	3.883 (7)	137
C7′—H7′1···I2^ix^	0.98	3.19	4.039 (7)	146

Symmetry codes: (i) −*x*−1, −*y*−1, −*z*; (v) −*x*, −*y*−1, −*z*; (vi) *x*, *y*−1, *z*; (vii) *x*, *y*+1, *z*; (viii) −*x*+1, −*y*+1, −*z*+1; (ix) *x*+1, *y*+1, *z*.
